# Model demonstrates functional purpose of the nasal cycle

**DOI:** 10.1186/s12938-015-0034-4

**Published:** 2015-04-24

**Authors:** David E White, Jim Bartley, Roy J Nates

**Affiliations:** School of Engineering, Auckland University of Technology, Auckland, New Zealand; Department of Surgery, University of Auckland, Auckland, New Zealand

**Keywords:** Nasal cycle, Airway surface liquid, Airflow partitioning, Air-conditioning model, Mucociliary transport

## Abstract

**Background:**

Despite the occurrence of the nasal cycle being well documented, the functional purpose of this phenomenon is not well understood. This investigation seeks to better understand the physiological objective of the nasal cycle in terms of airway health through the use of a computational nasal air-conditioning model.

**Method:**

A new state-variable heat and water mass transfer model is developed to predict airway surface liquid (ASL) hydration status within each nasal airway. Nasal geometry, based on *in-vivo* magnetic resonance imaging (MRI) data is used to apportion inter-nasal air flow.

**Results:**

The results demonstrate that the airway conducting the majority of the airflow also experiences a degree of ASL dehydration, as a consequence of undertaking the bulk of the heat and water mass transfer duties. In contrast, the reduced air conditioning demand within the other airway allows its ASL layer to remain sufficiently hydrated so as to support continuous mucociliary clearance.

**Conclusions:**

It is quantitatively demonstrated in this work how the nasal cycle enables the upper airway to accommodate the contrasting roles of air conditioning and the removal of entrapped contaminants through fluctuation in airflow partitioning between each airway.

## Background

All mammals, including man, have two nasal passageways which typically carry a differing apportionment of tidal airflow [1]. Periodic change in inter-nasal airflow apportionment is known as the nasal cycle. In healthy humans, the nose is the preferred entry point for air entering the airways [2-5], serving an important role in maintaining airway health by entrapping inhaled pathogens and pollutants as well as heating and humidifying inhaled air [6,7]. During nasal breathing, the nose recovers around 30% of exhaled heat and water vapour [8] and provides a region for olfaction to occur [9]. The entire conducting airway is lined with an airway surface liquid (ASL) that not only provides the means of entrapment of inhaled pathogens, but is also the medium through which heat and water must pass though from the underlying mucosa [10].

Currently it is thought that the nasal cycle controls the balance between heat and water fluxes from the ASL [3]. Other sources believe it enables cells and glands to rest and recharge [11]. No known definitive work in the current literature analyses or confirms these ideas.

Gaining transducer access along the complete nasal cavity is not practical without altering the complex geometry or eliciting a tissue reaction. For this reason, computational modelling is a useful manner to predict conditions within the nose. While some physical and computational nasal air conditioning models have been developed [12-17], none appear to have considered the complete nasal airway or the effect of the ‘nasal cycle’ on ASL hydration levels. This works seeks to remedy this through the incorporation of airflow apportionment as a result of the nasal cycle into a computational nasal air-conditioning model, which demonstrates new insights into this physiological phenomenon. This work also introduces for the first time the variable of ASL water equivalent height (H_e,asl_) which quantifies the change in ASL hydration status as a combination of the different hydration responses of its binary layers to water loss or gain.

### Mucociliary clearance

The defensive mucus layer within the nose is normally transported in the posterior direction at around 3–25 mm/min by the synchronized beating of motile cilia protruding from pseudo-stratified columnar epithelium [18,19]. This propels the upper mucus layer toward the pharynx where it is cleared by swallowing or expectoration.

### ASL hydration/dehydration

The nose is located at the opening of the conducting airway so its mucosa is exposed to a significantly greater air-conditioning demand than that encountered when moving toward the distal airways [12]. While many studies into maximal ASL supply have been undertaken for the trachea and bronchi [20-25], it is not possible to use those data for the nose model used in this investigation since the number of submucosal gland openings varies throughout the airway [26].

The ASL lining the entire conducting airway consists of two functional layers; the periciliary layer (PCL) overlaying the airway mucosa and the sticky mucus gel blanket facing the airway lumen. The PCL provides a platform for mucus transportation through motile cilia beating [24,27,28] while the sticky mucus layer has the important role of entrapment of inhaled pathogens/particles and the absorption of gaseous water-soluble air contaminants [20,29-31]. Recently this stratification and its implications for ASL hydration have been further clarified [32]. The gel-on-brush model recently proposed [32] describes how the mucins and mucopolysaccharides released from the mucosa become either tethered to the cilia to form a brush like structure, or alternatively, remain un-grafted and move into the mucus layer. The resultant variation in mucin density suggested by this model helps explain its stratification and, more importantly, also indicates why each ASL layer has a differing hydration behaviour. This variation is quantified in terms of osmotic bulk modulus and it serves to protect the PCL from significant height change over the normal ASL hydration range to ensure efficient mucociliary transport can be maintained [33,34]. Rehydration of the ASL follows through mucosal glandular [20] and purinergic supply channels [35]. During exhalation, supplementary rehydration also occurs as a consequence of mucosal cooling during inhalation. This enables the fully saturated, warm, exhaled air (at near core body temperature) to condense on the cooler ASL. Any excess ASL water content is reabsorbed through the airway epithelium [20]. Summarising the ASL hydration/height interplay [32]:

1. During normal ASL hydration/dehydration, water preferentially enters or leaves the mucus layer, causing it to undergo significant changes in height. The PCL layer remains at a relatively constant height of 7–10 μm, ensuring mucociliary transport is maintained.
2. Severe ASL dehydration causes high levels of water to be drawn from both mucus and PCL layers which causes significant reduction in PCL height.

During severe ASL dehydration, a reduction in PCL height causes mucociliary transport dysfunction [34,36]. Although it has not been investigated, it has been hypothesised that the likely mechanisms causing this may be mucus filling the interciliary spaces, or through the mucus layer compressing the motile cilia [32].

### Nasal cycle

It is reported that a proportion of the population, ranging from 20 – 40 % [1,37,38] to over 80% [1,39-41], experience periodic vascular congestion/decongestion of the erectile tissue within either side of the nose [41,42]. One airway demonstrates enlarged turbinates, obstructing airflow, while those in the other passageway are contracted. The airway that offers less obstruction to airflow is termed ‘patent’ while the other is referred to as being ‘congested’. The ‘nasal cycle’ describes the alternating patent and congested status of each nasal airway for periods ranging from 1 to 7 hours [43]. The span of the nasal cycle is made up from combinations of discrete ultradian periods spanning 1-1½ hours [39] and usually goes unnoticed since the total nasal airflow resistance remains unchanged [9,11]. While the most apparent outcome of this cycle is that it serves to regulate the bias of air mass flow partitioning between the airways [1,44], the functional purpose of the nasal cycle is not fully understood. Currently it is thought to control the balance between heat and water fluxes from the ASL [3], as well as enable cells and glands on the congested side to rest and recharge [11], but no definitive work in the literature analyses or confirms these ideas. Mucociliary transport is also faster within the congested airway [40], however the reasons for this are currently unknown.

## Methods

A new state-variable nasal air-conditioning model is used to predict ASL hydration status during ambient air breathing. This model predicts change in state variables of air temperature, *T*_*a*_*(x,t)*, and air water vapour concentration, *C*_*a*_*(x,t)*, along the nose as a function of both distance, *x* (a spatial dimension) and time, *t* (a temporal dimension). Although the blood temperature distribution and core computational method of this model follows that previously used by Hanna *et al.* [12,45], it differs significantly in several aspects including:

1. ASL hydration status is based on the recent ‘gel-on-brush’ model proposed by Button *et al*. [32].
2. Mucosal ASL water supply and reabsorption based on physiological data [46,^47^].
3. Calculation of state variables is undertaken over a complete breathing cycle.
4. Nasal geometry based on subject specific *in-vivo* magnetic resonance imaging (MRI) data [48].
5. Apportionment of inter-nasal air mass flow based on nasal airflow resistance.
6. Determination of ASL hydration status is undertaken along both nasal airways.

Additionally, heat and water mass transfer coefficients are calculated at small incremental distances along the airway [46], rather than interpolating the sparse empirical data obtained from cadaver studies [12,45]. For brevity, previous modelling work is not reviewed; rather, emphasis is given to describing the new attributes built into the model.

### Development of ASL water equivalent height

The parameter ASL water equivalent height (*H*_*e,ASL*_) is now introduced as a means of quantifying the change in ASL hydration status in terms of water equivalent loss from both ASL layers. To aid the explanation of this concept, *H*_*e,ASL*_ simply represents the equivalent water volume, in terms of height per unit area, removed from the normally hydrated binary layered ASL, to achieve a state of severe dehydration. In this state the ASL is characterised as being sufficiently dehydrated to cause cessation of mucociliary transport. Here, an 85% water volume reduction within the ASL causes the PCL layer to vary by 53% or 4 μm in height [32]. Approximating the osmotic bulk modulus of the PCL as being twice that of the mucus means the PCL layer experiences half the water loss as that of the mucus layer over the same dehydration range. Over these conditions, the change in mucus layer height (8 μm) is twice that experienced by the PCL (4 μm) to yield a total ASL height variation of 12 μm. Equating this to an 85% water volume loss between fully hydrated and dehydrated states results in *H*_*e,ASL*_ changing by 10 μm. Within the model we consider fully hydrated ASL to have an *H*_*e,ASL*_ of 10 μm, and when severely dehydrated, *H*_*e,ASL*_ = 0 μm.

### ASL supply

Water flux data, previously calculated from the difference between inhaled and nasopharynx air conditions, breathing volume and rate [9], is used to determine ASL water supply. Assuming dry inhaled air is fully humidified after passing down one nasal passageway of known typical surface area (100 cm^2^) [48] in one breath cycle enables the maximal ASL supply value for the nasal mucosa be estimated at 7.9 g/cm^2^-hr. The maximum rate of excess ASL absorption within the nose can now be determined by dividing this value by a reabsorption ratio. This ratio, calculated from earlier ASL supply and volume reduction investigations [20,21,49-51], is approximated at 3.5. This yields a maximum ASL reabsorption value of 2.2 g/cm^2^.hr. These ASL supply and reabsorption values are implemented within the new computational model.

While it is not possible to account for all possible variations in tidal breathing volume and frequency, for the purposes of this work, the tidal air mass flow, *m*_*a*_*(t)*, measured from an adult nasal breathing at rest [52], were implemented within the model.

### Nasal geometry

This model considers the two complex nasal cavities as a series (*k*) of aligned tubes of varying hydraulic diameter as a resistance network that provides two parallel airflow paths between the nares and nasopharynx [46]. Geometric analysis of each airway is calculated from an individual’s MRI *in-vivo* data, obtained from the group’s previous work [48], and analysed at discrete segments, or ‘lumps’, along each airway. Local nasal geometric parameters of cross-sectional area, *A(k),* and perimeter, *P(k)* were acquired from MRI slice thickness of 0.78 mm normal to the head coronal plane. These were used to determine local hydraulic diameter, *Dh(k)*, and airflow conditions of Reynolds number, Re_d_(*k*),.

### Inter-nasal airflow partitioning

Prediction of inter-nasal resistance is significant to the model since local air velocities, geometries and resultant airflow conditions determine the local pressure drop throughout the nose. Assuming at-rest breathing results in laminar airflow occurring throughout the nose simplifies the determination of air mass flow resistance within each airway. Previous investigations have found that laminar flow occurs throughout the nose during peak at-rest breathing [53,54]. These conditions were adopted for this work which simplifies the determination of air flow resistance, ƒ*(k)*. As the nasal passageways consist of a series of folded narrow-gap regions, it was decided to model local airflow resistance as equivalent to two closely spaced plates of infinite length to which an empirical relationship for ƒ*(k)* exists [55]. This assumption of parallel plates is justified by the distance between the opposing airway walls in each airway not exceeding 3 mm. Local ƒ*(k)* is described as a function of local airflow conditions, determined from Re_*d*_*(k),* given by equation (1)

1 $$ f(k)=\frac{24}{{\mathrm{Re}}_d(k)}, $$

The Darcy-Weisbach equation [55] is used to determine the pressure head loss, hƒ(*k*), for ducted flows using a characteristic airway dimension, *l*, local flow velocity, *V*, and gravity, *g*, as follows:

2 $$ \mathrm{h}f(k)=\frac{f(k)l{V}^2}{2gDh(k)} $$

Substituting equation (1) into (2) and considering fractional air mass flow within each airway, *m*_*a*_*(k)*, enables the local specific airflow resistance, *R(k)* to be calculated:

3 $$ R(k)=\frac{h_f(k)}{{\dot{m}}_a(k)}=\frac{3\mu P{(k)}^2l}{4{\rho}^2A{(k)}^3g}, $$

Here the local airway resistance, *R(k),* is expressed in terms of pressure head loss, *h*_*f*_*(k*), per unit air mass flow, ṁ_a_(*k*)_,_ over characteristic length, *l*. The total resistance of each airway, *R*_*total*_, is found by summing the resistance of all sections along each airway. This is then used to determine the total nasal resistance of both airways by considering the airways as a parallel resistance network [55,56], given by:

4 $$ \frac{1}{R_{total}}=\frac{1}{{{\displaystyle \sum R}}_{left}}+\frac{1}{{{\displaystyle \sum R}}_{right}}. $$

Calculation of the nasal airflow partitioning ratio (*PR*) for each airway is based on the ratio of total and individual airway airflow resistance and is based on unity airflow occurring within each airway. While in reality different airflows occur along each airway, this technique enables the tidal airflow to be apportioned through each airway as a function of their relative airflow resistances. The air mass flow partitioning ratio (*PR*) for each airway is now determined using a parallel resistance analysis of the whole nasal airway. The ratio of total airway resistance for the left and right airways respectively is given by:

5 $$ P{R}_{left}=\frac{R_{total}}{{{\displaystyle \sum R}}_{left}} $$

6 $$ P{R}_{right}=\frac{R_{total}}{{{\displaystyle \sum R}}_{right}} $$

Airflow through each nasal passageway was calculated by multiplying the instantaneous tidal breath mass flow rate by the respective left or right air mass flow partitioning ratio.

## Results

**Figure 1:**
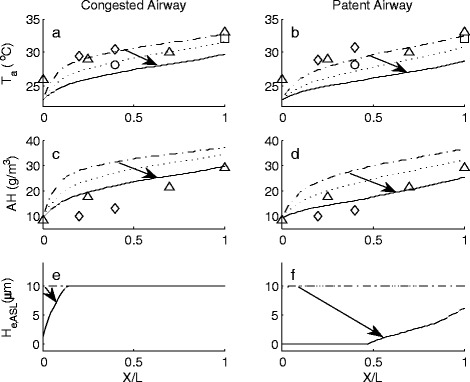
Inter-airway temperature (*T*_*a*_), absolute humidity (*AH*) and ASL water equivalent height (*H*_*e,ASL*_) distribution from rest to maximal change during inhalation of ambient air at *T* = 23°C, *RH* = 45% (*AH* = 9.2g H_2_O/m^3^ dry air). Arrows indicate direction of change. Non-dimensional airway position (X/L), defines the ratio of distance from anterior nasal valve to the posterior choanae within the two airways.  = commencement of inhalation,  = intermediate value,  = maximal reduction during inhalation. ∆ = Keck et al. [7], ○ = Wiesmiller et al. [57], ◊ = Lindemann et al. [58], □ = Lindemann et al. [59].

**Figure 2:**
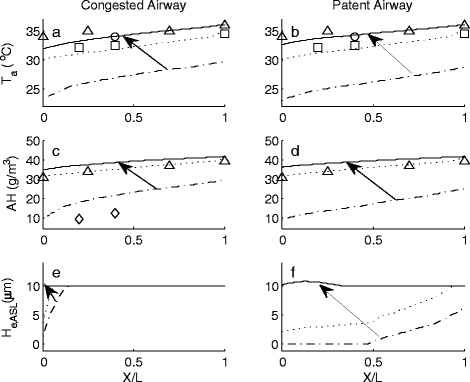
Inter-airway temperature (Ta), absolute humidity (AH) and ASL water equivalent height (He,ASL) distribution from end of inhalation to maximal change during exhalation. Arrows indicate direction of change. Non-dimensional airway position (X/L), defines the ratio of distance from anterior nasal valve to the posterior choanae within the two airways.  = commencement of exhalation,  = intermediate value,  = maximal increase during exhalation. ∆ = Keck et al. [7], ○ = Wiesmiller et al. [57], □ = Lindemann et al. [59].

Model predictions for inter-nasal air temperature (T_a_), absolute humidity (AH) and H_e,ASL_ distributions during the inhalation and exhalation phases of the breath cycle are shown in Figures 1 and 2 respectively. Non-dimensional airway position (X/L), defines the ratio of distance from anterior nasal valve to the posterior choanae within the two airways. This was used to account for variation in nasal passage length between MRI participant data sets. Figures 1 and 2 show the temperature, absolute humidity and ASL water equivalent height as a function of position within the airway and at different time instances during the breath cycle. These results highlight the continual variation in air temperature, water content as well as the ASL hydration state (not previously quantified) along each airway. The best way to interpret these results is to visualise them as an animation as it gives sense to the rate of change in these parameters during normal tidal breathing. To enable representation of results within this work, data is presented as temporal slices during the inhalation phase of the breath cycle (Figure 1). Here, results span the time interval of commencement of inhalation (depicted by the dash-dot line) to where maximal variation in measured parameters occur (solid line). Data corresponding to an intermediate interval (dotted line) are also shown. During expiration, restoration of normal ASL height occurs as a result of condensation of exhaled water vapour that re-hydrates the airway surfaces (Figures 2e,f). The previously dehydrated anterior region (X/L < 0.48) of the patent airway rehydrates due to the combination of water condensing from the saturated exhaled air and mucosal supply. Of note is slight over-hydration within the anterior region (X/L < 0.3) of this airway due to excess condensation occurring as a result of this passageway carrying a greater portion of exhaled saturated air mass flow when compared to the congested airway.

The arrows in the figures demonstrate the direction of change in each parameter over the inhalation and exhalation phases. These start from the data found at the beginning of each breath phase and point to the maximal changes that occur. Also included in these graphs are previously reported measured clinical data [7,57-59].

## Discussion

Model results for air temperature and absolute humidity during both inhalation and exhalation phases of breathing compare favourably with previous measured clinical data [7,57-59].

### Inhalation breath phase

Of note is the slight reduction in air temperature distribution along the patent airway, Figure 1b, when compared to the congested airway, Figure 1a. This is due to the higher air velocities occurring within the patent airway. This factor also influences the AH distribution along the patent airway; Figure 1d, which also demonstrates a lower value than that found in the congested airway, Figure 1c.

Model predictions for ASL hydration during peak inhalation indicate how the nose copes with its dual roles of air-conditioning and providing mucociliary clearance. Severe ASL dehydration is predicted to occur along 48% of the anterior nasal region of the patent airway (Figure 1f) as a consequence of this airway carrying the greater apportionment (67% in this case) of the air mass flow. In contrast, the congested airway experiences minor dehydration (Figure 1e) which does not exceed the ASL hydration limits, as a result of carrying a lesser air mass flow. While variation in the breath cycle, in terms of amplitude or period, or alteration in ambient air conditions, would cause change in the severity and extent of ASL dehydration, these would not change the characteristic difference in ASL hydration behaviour between either of the two airways.

Previous investigations into the nasal cycle have found that that the mucociliary transport velocity within the congested airway is greater than that found in the patent airway [40]. These findings support the model results, which demonstrate for the first time that the patent airway experiences recurring severe ASL dehydration during inhalation while the ASL within the congested airway remain sufficiently hydrated so as to support continuous mucociliary transport.

The finding of severe ASL dehydration that causes reduced mucociliary transport occurring only within the patent airway is new. The body manages this negative outcome by periodically switching the bias of air mass-flow partitioning between the two nasal airways. This change in airflow apportionment is known as the nasal cycle and it enables efficient and sustained mucociliary transport to commence within the previously patent airway. On the other hand, the previously congested airway, after having a period of rest and recovery, and efficient mucociliary clearance, then experiences ASL dehydration and resultant poor mucociliary clearance during inhalation. Change in nasal cycle status also limits the exposure time each airway has to endure these adverse ASL drying conditions.

### Exhalation breath phase

Figure 2f demonstrates that the ASL within the patent airway becomes fully hydrated by completion of the exhalation phase. The congested airway (Figure 2e), which did not become dehydrated during inhalation, remains at the normal physiological maximal limit.

### Explanation of the nasal cycle

The use of the air-conditioning model gives greater insight into the functional purposes of the nasal cycle. The ability to efficiently transport entrapped inhaled pathogens and pollutants requires low air velocities and sustained ASL hydration, whilst the role of air-conditioning requires high air velocities to be effective. Model results demonstrate that cyclic ASL dehydration occurs in the patent airway during inhalation in response to air-conditioning demands during tidal breathing. Both air conditioning and filtration duties are predominantly carried within the anterior nasal region and must be simultaneously and continuously carried out in order to maintain airway health. The nose copes with this conflict by regulating inter-nasal air mass-flow through change in the nasal cycle. This results in the previously patent airway, stressed from carrying the bulk of the heat and water mass transfer duties and experiencing issues associated with severe ASL dehydration, becoming congested. Switching to this state enables its ASL layer to return to an uninterrupted state of hydration that supports continuous and normal mucociliary clearance. On the other hand, the previously congested airway, having had experienced a period of cellular rest and recovery, picks up the bulk of the heat and water mass transfer duties and, for a limited time, endures issues associated with cyclic severe ASL dehydration. This balancing of heat and water fluxes between airways is consistent with the current understanding of the purpose of the nasal cycle to control the balance between heat and water fluxes from the ASL [3], as well as enable cells and glands on the congested side to rest and recharge [11]. The new suggestion from this work is that that it is also the way in which the anterior conducting airway copes with its multiple duties that require conflicting characteristics of high and low air flow velocities. It is proposed that the nasal cycle, characterised by recurrent variation in air mass-flow partitioning between each airway, enables each passageway to alternatively take turns in either predominantly undertaking the air-conditioning or mucus clearance roles.

## Conclusions

While the nasal airways in all healthy mammals, including man, demonstrate the nasal cycle, the reason for phenomenon has previously not been well understood. Early work by Eccles *et al.* (1982) proposed that the ‘nasal cycle’ enables cells and glands to rest and recharge [11]. Later work [3] has hinted that the ‘nasal cycle is probably controlling the balance between the fluxes of heat and water vapour required to condition the inspired air and the ability of nasal blood flow and mucus secretion to supply sufficient heat and water to the surface tissue surface’. Our finding support both of these views and also demonstrates that the nasal cycle provides a means by which the anterior conducting airway copes with conflicting ASL hydration states where each passageway alternatively take turns in either predominantly undertaking the air-conditioning or mucus clearance roles. The model also provides a means of tracking the time and space-variable changes of conditions in the individual nasal passageways and therefore further studying the physiological response to these variations. This discovery re-ignites interest in an often overlooked physiological phenomenon.

## References

[1] Hanif J, Jawad SSM, Eccles R (2000). The nasal cycle in health and disease. Clin Otolaryngol.

[2] Bartley J (2006). Breathing matters: a New Zealand guide.

[3] Elad D, Wolf M, Keck T (2008). Air-conditioning in the human nasal cavity. Respir Physiol Neurobiol.

[4] Naftali S, Rosenfeld M, Wolf M, Elad D (2005). The air-conditioning capacity of the human nose. Ann Biomed Eng.

[5] Chhabra N, Houser SM (2009). The diagnosis and management of empty nose syndrome. Otolaryngol Clin North Am.

[6] Drettner B, Falck B, Simon H (1977). Measurements of the Air conditioning capacity of the nose during normal and pathological conditions and pharmacological influence. Acta Otolaryngol (Stockh).

[7] Keck T, Leiacker R, Heinrich A, Kuhnemann S, Rettinger G (2000). Humidity and temperature profile in the nasal cavity. Rhinology.

[8] Cole P, Proctor DF, Andersen I (1982). Modification of inspired air. The nose: upper airway physiology and the atmospheric environment.

[9] Wolf M, Naftali S, Schroter RC, Elad D (2004). Air-conditioning characteristics of the human nose. J Laryngol Otol.

[10] Warren N, Crampin E, Tawhai M (2010). The role of airway epithelium in replenishment of evaporated airway surface liquid from the human conducting airways. Ann Biomed Eng.

[11] Eccles R, Proctor DF, Andersen I (1982). Neurological and pharmacological considerations. The nose: upper airway physiology and the atmospheric environment.

[12] Hanna LM, Scherer PW (1986). Regional control of local airway heat and water vapor losses. J Appl Physiol.

[13] Naftali S, Schroter RC, Shiner RJ, Elad D (1998). Transport phenomena in the human nasal cavity: a computational model. Ann Biomed Eng.

[14] Fodil R, Brugel-Ribere L, Croce C, Sbirlea-Apiou G, Larger C, Papon J-F (2005). Inspiratory flow in the nose: a model coupling flow and vasoerectile tissue distensibility. J Appl Physiol.

[15] Hahn I (1992). Modeling nasal airflow and olfactory mass transport.

[16] Keyhani K (1995). Numerical modeling of airflow and mass transfer in the human nasal cavity.

[17] Lee HP, Poh HJ, Chong FH, Wang DY (2009). Changes of airflow pattern in inferior turbinate hypertrophy: a computational fluid dynamics model. Am J Rhinol Allergy.

[18] Mygind N, Dahl R (1998). Anatomy, physiology and function of the nasal cavities in health and disease. Adv Drug Delivery Rev.

[19] Boek WM, Graamans K, Natzijl H, van Rijk PP, Huizing EH (2002). Nasal mucociliary transport: new evidence for a key role of ciliary beat frequency. Laryngoscope.

[20] Widdicombe JH (2002). Regulation of the depth and composition of airway surface liquid. J Anat.

[21] Widdicombe JH, Bastacky SJ, Wu DX, Lee CY (1997). Regulation of depth and composition of airway surface liquid. Eur Respir J.

[22] Wu DXY, Lee CYC, Uyekubo SN, Choi HK, Bastacky SJ, Widdicombe JH (1998). Regulation of the depth of surface liquid in bovine trachea. Am J Physiol Lung Cell Mol Physiol.

[23] Ballard ST, Trout L, Bebök Z, Sorscher EJ, Crews A (1999). CFTR involvement in chloride, bicarbonate, and liquid secretion by airway submucosal glands. Am J Physiol Lung Cell Mol Physiol.

[24] Trout L, King M, Feng W, Inglis SK, Ballard ST (1998). Inhibition of airway liquid secretion and its effect on the physical properties of airway mucus. Am J Physiol Lung Cell Mol Physiol.

[25] Cho H-J, Joo NS, Wine JJ. Defective Fluid Secretion from Submucosal Glands of Nasal Turbinates from CFTR−/− and CFTR^ΔF508/ΔF508^ Pigs. PLoS ONE, 2011, 6(8): 1-11. 10.1371/journal.pone.0024424 PMC3164206 21935358

[26] Whimster WF (1986). Number and mean volume of individual submucous glands in the human tracheobronchial tree. Appl Pathol.

[27] Tarran R, Trout L, Donaldson SH, Boucher RC (2006). Soluble mediators, Not cilia, determine airway surface liquid volume in normal and cystic fibrosis superficial airway epithelia. J Gen Physiol.

[28] Button B, Boucher RC (2008). Role of mechanical stress in regulating airway surface hydration and mucus clearance rates. Respir Physiol Neurobiol.

[29] Cone RA (2009). Barrier properties of mucus. Adv Drug Delivery Rev.

[30] Bossi R, Piatti G, Roma E, Ambrosetti U (2004). Effects of long-term nasal continuous positive airway pressure therapy on morphology, function, and mucociliary clearance of nasal epithelium in patients with obstructive sleep apnea syndrome. Laryngoscope.

[31] Winther B, Gwaltney JR, Kennedy DW, Bolger WE, Zinreich SJ (2001). Microbiology of sinusitis. Diseases of the sinuses - diagnosis and management.

[32] Button B, Cai L-H, Ehre C, Kesimer M, Hill DB, Sheehan JK (2012). A periciliary brush promotes the lung health by separating the mucus layer from airway epithelia. Science.

[33] Refojo MF (1975). Vapor pressure and swelling pressure of hyrdogels. American Chemical Society, Polymer Preprints, Division of Polymer Chemistry.

[34] Quraishi MS, Jones NS, Mason J (1998). The rheology of nasal mucus: a review. Clin Otolaryngol.

[35] Tarran R, Button B, Boucher RC (2006). Regulaton of normal and cystic fibrosis airway surface liquid volume by phasic shear stress. Annu Rev Physiol.

[36] Williams RB, Rankin N, Smith T, Galler D, Seakins P (1996). Relationship between the humidity and temperature of inspired Gas and the function of the airway mucosa. Crit Care Med.

[37] Davis SS, Eccles R (2004). Nasal congestion: mechanisms, measurement and medications. Core information for the clinician Clin Otolaryngol.

[38] Hasegawa M, Ohki M, Kurita N (1990). Effects of posture on the nasal cycle. Am J Rhinol.

[39] Atanasov AI, Dimov PD, Dimitrov BD (2003). Time periods in the nasal cycle during night sleep. Biol Rhythm Res.

[40] Littlejohn, M.C., et al., The relationship between the nasal cycle and mucociliary clearance. The Laryngoscope, 1992. 102(2): p. 117-120. 10.1288/00005537-199202000-00002 1738280

[41] Lindemann J, Leiacker R, Rettinger G, Keck T (2003). The relationship between water vapour saturation of inhaled air and nasal patency. Eur Respir J.

[42] Druce HM (1988). Measurement of nasal mucosal blood flow. J Allergy Clin Immunol.

[43] Kennedy DW, Zinreich SJ, Kumar AJ, Rosenbaum AE, Johns ME (1988). Physiologic mucosal changes within the nose and ethmoid sinus: imaging of the nasal cycle by MRI. Laryngoscope.

[44] Roblin DG, Eccles R (2003). Normal range for nasal partitioning of airflow determined by nasal spirometry in 100 healthy subjects. Am J Rhinol.

[45] Hanna LM (1983). Modelling of heat and water vapor transport in the human respiratory tract (air-conditioning).

[46] White DE (2013). Nasal drying during pressurised breathing.

[47] White D, Nates R, Bartley J (2014). A pilot study of an in-vitro bovine trachea model of the effect of continuous positive airway pressure breathing on airway surface liquid. Biomed Eng Online.

[48] White DE, Al-Jumaily AM, Bartley J, Lu J (2011). Correlation of nasal morphology to air-conditioning and clearance function. Respir Physiol Neurobiol.

[49] Cheng YS, Yeh HC, Guilmette RA, Simpson SQ, Cheng KH, Swift DL (1996). Nasal deposition of ultrafine particles in human volunteers and its relationship to airway geometry. Aerosol Sci Technol.

[50] Jiang C, Finkbeiner WE, Widdicombe JH, McCray PB, Miller SS (1993). Altered fluid transport across airway epithelium in cystic fibrosis. Science.

[51] Button B, Picher M, Boucher RC (2007). Differential effects of cyclic and constant stress on ATP release and mucociliary transport by human airway epithelia. J Physiol.

[52] White DE (2003). Breathing therapy air delivery unit: simulation, design and development.

[53] Guan-xia X, Jie-Min Z, Hong-Yan J, Jian-Feng L, Liang-Wan R, Gen X (2008). Computational fluid dynamics simulation of airflow in the normal nasal cavity and paranasal sinuses. Am J Rhinol.

[54] Hörschler I, Schröder W, Meinke M (2010). On the assumption of steadiness of nasal cavity flow. J Biomech.

[55] White FM (1979). Fluid mechanics.

[56] Douglas JF, Gasiorek JM, Swaffield JA (1995). Fluid mechanics.

[57] Wiesmiller K, Keck T, Leiacker R, Lindemann J (2007). Simultaneous in vivo measurements of intranasal air and mucosal temperature. Eur Arch Otorhinolaryngol.

[58] Lindemann J, Leiacker R, Stehmer V, Rettinger G, Keck T (2001). Intranasal temperature and humidity profile in patients with nasal septal perforation before and after surgical closure. Clin Otolaryngol Allied Sci.

[59] Lindemann J, Leiacker R, Rettinger G, Keck T (2002). Nasal mucosal temperature during respiration. Clin Otolaryngol.

